# Advances about the Roles of Membranes in Cotton Fiber Development

**DOI:** 10.3390/membranes11070471

**Published:** 2021-06-25

**Authors:** Fan Xu, Qian Chen, Li Huang, Ming Luo

**Affiliations:** 1 Biotechnology Research Center, Key Laboratory of Biotechnology and Crop Quality Improvement of Ministry of Agriculture, Southwest University, Chongqing 400715, China; xufanfeiren@163.com (F.X.); hl19970914@email.swu.edu.cn (L.H.); 2 College of Horticulture and Landscape Architecture, Southwest University, Chongqing 400715, China; chenqiansuaige@163.com

**Keywords:** membrane, cotton, fiber cells

## Abstract

Cotton fiber is an extremely elongated single cell derived from the ovule epidermis and is an ideal model for studying cell development. The plasma membrane is tremendously expanded and accompanied by the coordination of various physiological and biochemical activities on the membrane, one of the three major systems of a eukaryotic cell. This review compiles the recent progress and advances for the roles of the membrane in cotton fiber development: the functions of membrane lipids, especially the fatty acids, sphingolipids, and phytosterols; membrane channels, including aquaporins, the ATP-binding cassette (ABC) transporters, vacuolar invertase, and plasmodesmata; and the regulation mechanism of membrane proteins, such as membrane binding enzymes, annexins, and receptor-like kinases.

## 1. Introduction

Cotton (*G. hirsutum* L.) is the world’s most important natural fiber for the textile industry and a mainstays of the global economy [1]. This single-celled fiber, formed by polar elongation and the secondary cell wall (SCW) thickening of the ovule epidermal cell, makes it an ideal material for studying cell elongation and SCW deposition [2,3]. Cell development passes through four stages: initiation, elongation, secondary wall synthesis, and mature dehydration. The initiation, elongation, and SCW deposition periods determine the length, strength and fineness of the fiber [4], which requires the coordination of cell wall yield properties, turgor pressure, lipid biosynthesis, and cell wall components and proteins [1].

The plasma membrane (PM) surrounding each cell serves as an active communication interface between the cell, neighboring cells, and eventually the whole organism [5]. The membrane a remarkably complex organization of lipids and proteins [6]. The lipids consist of phospholipids, sphingolipids and sterols, of which the phospholipids are the main components of the lipid bilayer [7,8], while sphingolipids and sterols are enriched in microdomains in the PM, also called membrane lipid raft [9], and their predominant role is to maintain its structural integrity. Recently, they have been thought to act as signaling molecules in several processes including programmed cell death (PCD) [10,11] and responses to biotic and abiotic stress [9]. Proteins embedded in the membrane lipid bilayer (ion channels) are essential tunnels and dynamic regulators of ion flux across membranes. The function of ion channels in regulating osmotic pressure, growth, signaling, movement, and nutrient acquisition has been fully reviewed by [12]. Plasmodesmata (PDs) are tight membrane contact sites and channels that extend through the cell wall to establish both membrane and cytosolic continuity and serve as conduits for the transport of proteins, small RNAs, hormones, and metabolites during development and defense signaling. They also play essential roles in controlling cell-to-cell connectivity [13].

Plant PM-resident receptors recognize exogenous and endogenous signals, and then trigger proper responses to ensure a balanced modulation of development and stress adaptation [14,15]. Heterotrimeric G-proteins have crucial roles in regulating signaling pathways that are essential for growth and development [16]. The Rop guanosine triphosphatases (GTPases) of small GTP-binding superfamily proteins regulate cell expansion through cortical actin/microtubule dynamics [17]. The cell walls provide support and protection and determine both plant morphology and mechanical characteristics. The PM-localized cellulose synthase (CESA) complex contains essential enzymes for cellulose synthesis, which, together with proteins and other biomolecules, make up the cell walls [18,19].

Over the past two decades, many advances have been made concerning the roles of membrane functions in cotton fiber development. In this review, we summarize this research from three aspects: the functions of membrane lipids in cotton fiber growth, the roles of membrane channels in cotton fiber development, and the regulation mechanism of membrane proteins in fiber development.

## 2. The Functions of Membrane Lipids in Cotton Fiber Development

Fatty acids and lipids, which are essential constituents of all plant cells, not only provide structural integrity and energy for various metabolic processes but also function as signal transduction mediators [20]. Transcriptome analysis revealed that, during fiber cell elongation, lipid metabolism pathways are significantly up-regulated [21]. Cold stress is related to membrane structure; however, the relationship between cold stress and fiber needs to be strongly confirmed, but the formation of unsaturated fatty acids under cold stress could maintain the specific membrane structure required for fiber elongation [22]. Fiber cells contain significantly higher amounts of phosphatidylinositol (PI) with PI 34:3 being the most predominant species. The fatty acid desaturases, PI synthase, and PI kinase encoded genes (*Δ^15^GhFAD*, *PIS*, and *PIK*) were preferentially expressed in fibers. The application of linolenic acid (C18:3), soybean L-a-PI, and phosphatidylinositol monophosphate in cultured cotton ovules significantly promoted fiber growth, whereas a treatment with a liver PI lacking the C18:3 moiety, linoleic acid, and phosphatidylinositol monophosphate was completely ineffective. Moreover, suppression of *Δ^15^GhFAD*, *GhPIS*, and *GhPIK* resulted in significantly short-fibered phenotypes [23]. These evidence provide the basis for in-depth studies on the roles of PM-lipids in mediating cotton fiber growth.

The lipid raft is a microregion in the membrane lipid bilayer composed mainly of sphingolipids, sterols and proteins, and is an important part of the regulatory center of the membrane [5]. Sphingolipids are complex soluble fats that are found in all animals, plants, fungi and in few prokaryotes and viruses. The sphingolipid molecule has three main components: a long chain base (LCB), a long chain fatty acids (LCFA) or a very long chain fatty acid (VLCFA), and a polar head group. Sphingolipids play important roles in signaling pathways and many cellular processes, such as membrane protein targeting [24,25]. Fumonisin B1 (FB1) is produced by *Fusarium moniliforme*, which has a structure very similar to that of sphinganine (Sph) and acts as a specific inhibitor of ceramide synthase (CS) [26]. Wang et al., reported that FB1 severely blocked fiber cell elongation in cultured cotton ovules. Sphingolipidomic results of FB1 treated ovules showed that 95 sphingolipids were altered after FB1 treatment, of which 29 were significantly increased, while 33 were significantly decreased. Proteomic analysis found 633 upregulated and 672 downregulated proteins after FB1 treatment, and most of the differentially expressed proteins (DEPs) were involved in processes related to phenylpropanoid and flavonoid biosynthesis. Additionally, FB1 significantly suppressed the expression of plasmodesma callose-binding protein 3-like [27]. Exogenous application of saturated very-long-chain fatty acids (VLCFAs; C20:0 to C30:0) to ovules in in vitro cultures significantly promoted cotton fiber cell elongation, whereas those treated with VLCFA biosynthesis inhibitor, acetochlor (2-chloro-N-[ethoxymethyl]N-[2-ethyl-6-methyl-phenyl]-acetamide; ACE) abolished fiber growth. This inhibition was overcome by adding lignoceric acid (C24:0), which induced a rapid and significant increase in ACO (for 1-aminocyclopropane-1-carboxylic acid oxidase) transcript levels, resulting in substantial ethylene production and indicating that VLCFAs may act upstream of ethylene to promote fiber cell elongation [28]. These results indicated that sphingolipids have important roles in fiber elongation.

Plant sterols, also known as phytosterols, are an important component of membranes and a precursor of brassinosteroid (BR) biosynthesis. Sterols has been reported to play essential roles in cell elongation, microtubule cell development and arrangement, cellulose synthesis, and cell wall formation [29]. *GhDET2*, which encodes the steroid 5α-reductase, was highly expressed during the fiber initiation and rapid fiber elongation stages. Suppression of *GhDET2* inhibited both expressions, while seed coat-specific expression of *GhDET2* increased fiber number and length. These results indicated that the *GhDET2* gene plays an important role in the initiation and rapid elongation of cotton fibers [30]. The highest expression of the sterol C-24 methyltransferase gene, *GhSMT2-1*, was found at 10 DPA (days past anthesis), which was consistent with the period when the fiber cells had the highest elongation rate, and a high sterol content was found at the rapid fiber elongation stage. This showed that *GhSMT2-1* is important for fiber elongation [31]. The decrease or increase in the ratio of phytosterol/sitosterol was related to the promotion or inhibition of fiber elongation, respectively. Compared with wild-type TM-1, the expression of genes related to plant sterol biosynthesis was downregulated in the short fibers of the super-short fiber mutant *Li-1*. These results indicated that sterols are important for the development of cotton fiber cells, especially in the elongation of cotton fibers [32]. Niu et al. found that *GhSMT2-1* overexpression led to changes in phytosterol content and the campesterol to sitosterol ratio. At the rapid elongation stage, total phytosterol and sitosterol content increased while campesterol content was decreased in transgenic fibers when compared to control fibers. Accordingly, the ratio of campesterol to sitosterol declined strikingly. Simultaneously, the transgenic fibers were shorter and thicker than control fibers. Exogenous application of sitosterol or campesterol separately in vitro inhibited control fiber cell elongation in a cotton ovule culture system. In addition, campesterol treatment partially rescued transgenic fiber elongation. There might be a specific ratio of campesterol to sitosterol in different developmental stages of cotton fibers, in which *GhSMT2-1* plays an important role [33].

The molecular function of sterols in cellulose biosynthesis was well reviewed by Schrick et al. The function of the cellulose synthase complex requires a specific lipid environment, which may be provided by the lipid raft, a sphingolipid- and sterol-rich microdomain [34]. It was observed that the lipid raft coexists with other membrane fluid domains [5], so its activity was used to estimate membrane properties. In the fiber cell development, lipid raft activity exhibited a low–high–low change in regularity during fiber cell development, but the pattern was disrupted in the short-lint fiber *Ligon lintless-1 (Li1)* mutant, suggesting that membrane lipid order and lipid raft activity are closely linked to cell development [35].

## 3. The Role of Membrane Channels in Cotton Fiber Development

Cell expansion is a major component of plant cell development and organ growth. As a unidirectional cell expansion process, cotton fiber elongation is a result of the complex interplay between cell turgor and cell wall extensibility [36,37]. Aquaporins (AQPs) are membrane channels that facilitate the transport of water and small neutral molecules across biological membranes that belong to a highly conserved group of proteins called intrinsic proteins. AQPs play crucial roles in plant–water relations and cell turgor pressure maintenance and are required for growth and development and responses to multiple biotic and abiotic stresses [38,39]. By measuring levels of mRNA and protein accumulation and enzyme activity, it was possible to analyze the expression of components involved in turgor regulation: plasma membrane proton-translocating ATPase, vacuole-ATPase, proton-translocating pyrophosphatase (PPase), phosphoenolpyruvate carboxylase, and major intrinsic proteins. All but the PPase were highly accumulated during peak expansion (12–15 DPA), and then declined with the onset of secondary cell wall synthesis. The PPase was constitutively expressed through all development stages. Additionally, the activity of the two proton-translocating-ATPases peaked at 15 DPA, whereas PPase peaked at 20 DPA. These cues suggested that the turgor-related genes were regulated at the transcriptional and posttranslational levels through fiber development [36]. 

The plant aquaporins were divided into 5 subfamilies: plasma membrane intrinsic proteins (PIP), tonoplast intrinsic proteins (TIP), NOD26-like intrinsic proteins (NIP), small basic intrinsic proteins (SIP), and the recently discovered X intrinsic proteins (XIP). There were 71 aquaporin genes in upland cotton (*G. hirsutum*), and 28, 23, 12, 7, and 1 of them belonged to the 5 subfamilies, respectively [40]. Two cotton PIP/TIP encoding genes, *GhPIP1-2* and *GhcTIP1*, were predominantly expressed during fiber elongation, with the highest expression levels in 5 DPA fibers, implying that they might support the rapid influx of water into vacuoles during fiber cell expansion [41]. PIPs usually form hetero-oligomers to perform their functions. For cotton PIP2 groups, GhPIP2;3 interacted with GhPIP2;4 and GhPIP2;6, but GhPIP2;6 did not interact with GhPIP2;4. Co-expression of GhPIP2;3/2;4 or GhPIP2;3/2;6 resulted in an increased oocyte permeability coefficient. Overexpression of *GhPIP2* genes in yeast induced longitudinal growth, whereas down-regulation of *GhPIP2* genes in cotton markedly hindered fiber elongation. That is to say, GhPIP2 proteins selectively form hetero-oligomers to regulate their activities to meet the requirements for fiber elongation [42].

The ATP-binding cassette (ABC) transporters serves to translocate a broad range of substances across biological membranes powered by ATP hydrolysis [43]. *GhWBC1*, a cotton ABC transporter, was highly expressed in developing fiber cells and peaked in rapidly expanding fibers from 5 to 9 DPA. The overexpression of *GhWBC1* in *Arabidopsis* exhibited short siliques, implying that *GhWBC1* might have participated in cotton fiber elongation [44].

Vacuolar invertase (VIN) has long been known to be important for cell expansion [45]. Its activity during rapid elongation stage was approximately 4-6-fold of that in leaves, stems, and roots, and its activity in a genotype with faster fiber elongation was significantly higher than that in a slow-elongating genotype, implying that VIN plays a pivotal role in cotton fiber elongation. The expression of *GhVIN1* was closely matched by VIN activity and the fiber elongation rate. Ectopic expression of *GhVIN1* could complement the short-root phenotype of a VIN T-DNA mutant in *Arabidopsis*. Moreover, up- and downregulation of *GhVIN1* resulted in an increase or decrease in elongation, respectively [46,47]. Suppression of *GhVIN1* led to a fiberless phenotype in a dosage-dependent manner by regulating hexose signaling and the transcription of several MYB transcription factors and auxin signaling components required for fiber initiation [48]. This demonstrated the essential role of VIN in early fiber elongation.

Plasmodesmata (PD) are intercellular pores connecting most plant cells and controlling the entry and exit of molecules at cell boundaries [49]. The fiber PD were initially permeable at 0–9 DPA, closed at 10 DPA, and re-opened at 16 DPA. The expression of sucrose and K^+^ transporter genes were consistent with the transient closure of the PD and maximally in the 10 DPA fibers. Consequently, the osmotic and turgor potentials were elevated during this period, indicating that elongation was achieved largely by cell wall loosening and terminated by increased wall rigidity and loss of higher turgor [37]. Moreover, the PD closure positively correlated with fiber length among three tetraploid genotypes and two diploid progenitors. Additionally, the callose deposition and degradation at the fiber base correlated with the timing of PDs closure and reopening, respectively. The expression of the fiber-specific β-1,3-glucanase gene, *GhGluc1*, coincided with this pattern during fiber elongation, and was high in the short fiber genotype and weak in the intermediate- and long-fiber genotypes [50]; that is, the duration of the PD closure correlated positively with the final fiber length, which supports the idea that PD closure may be required for fibers to achieve extended elongation. Delayed expression of *Sus* in the seed-coat epidermis that correlates temporally and spatially with the initiation of fiber cells was observed in a lintless mutant *fls*. In addition, no closure of PD was visible during the entire elongation period of short fibres from this mutant, indicating that the short-fiber cell phenotype of the *fls* mutant correlated with the delayed or insufficient expression of *Sus* in a subset of seed-coat epidermal cells and their inability to close PD [51]. Suppressing the expression of the sterol carrier protein gene, *GhSCP2D* led to reduced sterol content and closed PD at 5 through 25 DPA. The abnormally closing of PD in *GhSCP2D* suppression lines was due to reduced expression of the PD-targeting β-1,3-glucanase *GhPdBG3-2A/D*. In addition, suppressing *GhSCP2D* upregulated a cohort of SUT and SWEET sucrose transporter genes in fiber cells [52]. This evidence indicated that PDs indeed play vital roles in fiber cell development.

## 4. The Regulation Mechanism of Membrane Proteins in Cotton Fiber Development

PM serves as the site of attachment of most enzymes, about 80% of which are membrane binding such as the cellulose synthase complex is located in the plasma membrane [35]. Sucrose synthase (SuSy) has always been considered to be a cytoplasmic enzyme, and in the development of cotton fiber, at least half of SuSy is tightly associated with the PM, which might channel carbon directly from sucrose to glucan implying that SuSy might have some role in cell wall synthesis [53]. Immunolocalization results showed that SuSy is localized in a proximal exoplasmic zone near cortical microtubules and PM, and the callose (β-1,3-glucan) was co-distributed with SuSy within this zone [54]. Expression analyses suggested that most SuSy genes had development-dependent expression profiles [55]. The *SusC* isoform of *SuSy* has been reported to be expressed at high levels during secondary cell wall synthesis [56]. Through transforming cotton with SuSy suppression constructs, Ruan et al. showed that the suppression of SuSy in the maternal seed tissue repressed fiber development, while suppression in the endosperm and embryo inhibited embryo development and seed size [57]. The expression of *GhSusA1* was significantly enhanced in GA-overproducing transgenic fibers and was induced by the exogenous application of bioactive GA in cultured fibers [58]. The suppression of *GhSusA1* led to reduced fiber quality, boll size, and seed weight, while the overexpression of this gene increased fiber length and strength [59]. In addition, the overexpression of a potato *SuSy* and a synthetic *SuSy* gene also improved cotton fiber quality [60,61]. Therefore, PM-associated SuSy are important for cotton fiber development, and could be used for improving fiber products.

SCW deposition is crucial for cotton fiber strength and fineness and a large amount of cellulose biosynthesis is mainly carried out to promote cell wall thickening [62]. Cellulose is synthesized by the PM-associated cellulose synthase complex (CSC) [63]. The cellulose synthase gene superfamily includes the cellulose synthase (Ces) and cellulose synthase-like (Csl) families. There were 228 Ces/Csl genes from four *Gossypium* species (*G. hirsutum*, *G. barbadense*, *G. arboreum*, and *G. raimondii*). Transcriptome analysis revealed that *CesA* genes were more highly expressed in tetraploids than in diploids, whereas *Csl* expression levels exhibited the opposite trend [64]. In addition, 18 *GhCesA* genes were located near the region of 74 quantitative trait loci associated with fiber quality [65]. The expression of *GhCesA4* was significantly upregulated at the SCW synthesis stage, and *GhCesA4* is important for cellulose biosynthesis during cotton fiber development [66]. *GhCesA2* was preferentially expressed during the cellulose biosynthesis stage of fiber development, and was highly expressed in one near-isogenic line (NIL) with higher fiber bundle strength, implying that *GhCesA2* was related to fiber strength [67]. Phylogenetic and gene co-expression analysis revealed that *GhCesA1*, *GhCesA2*, *GhCesA7*, and *GhCesA8* were mainly in charge of SCW biosynthesis, whereas *GhCesA3*, *GhCesA5*, *GhCesA6*, *GhCesA9*, and *GhCesA10* were involved in primary cell wall formation [68]. Overexpression of the *GhCSLD3* gene enhanced primary cell wall synthesis, resulting in restored cell elongation and cell wall integrity. It then partially rescued the growth defect of the *atcesa6* mutant during early vegetative growth [69]. These cues implied that the differential expression profile of genes associated with SCW cellulose biosynthesis was associated with cotton fiber properties. Recently, Zhang et al. resolved the structure of GhCesA7. Its homotrimer showed a C3 symmetrical assembly, and each protomer contained seven transmembrane helices (TMs), which formed a channel that facilitated the release of newly synthesized glucans. The cytoplasmic glycosyltransferase (GT) domain of GhCesA7 protruded from the membrane to form a catalytic pocket towards the TM pore that facilitated microfibril formation [70].

Cotton fiber annexins bind to the membranes in a Ca^2+^-dependent manner, and modulate the activity or localization of callose synthase [71]. *AnxGb6* was specifically expressed in elongating cotton fibers, and its expression correlated with cotton fiber length, especially the fiber elongation rate. Overexpression of *AnxGb6* in *Arabidopsis* enhanced root elongation without increasing the root cell number [72]. *AnnGh3*, *AnnGh4*, and *AnnGh5* were preferentially expressed in rapidly elongating fibers. Ectopic expression of *AnnGh3* in *Arabidopsis* resulted in a significant increase in trichome density and length of leaves [73]. AnxGb5/6 and their interacted proteins generated a protein macroraft in the cell membrane that was probably a stabilizing scaffold for Actin1 organization [74]. These results suggested that annexins may link Ca^2+^ signaling and actin assembling to the membrane to regulate fiber cell elongation.

As mentioned above, receptor-like kinases (RLKs) and receptor-like proteins (RLPs) on the PM are essential for mediating cell-to-cell and cell-to-environment communication, and then to regulate the balance between growth and immunity. Dynamic transcriptome analysis of the short fiber mutant *Li1* showed that common, differentially expressed genes (DEGs) were involved in the responses to auxin- and receptor kinase-related pathways for fibers bearing ovules at 3 and 8 DPA [75]. RNA sequencing of 15 and 20 DAP fiber cells from cotton lines MD52ne and MD90ne indicated that receptor-like kinases are potential candidate genes responsible for superior fiber strength in MD52ne [76]. Expression of genome-wide cotton *LRR-RLK* genes were involved in stress defense and diverse developmental processes, including fiber development [77]. The cotton genome has 29 wall-associated kinases (WAK), most of which are highly expressed in fibers and ovules [78]. *GhRLK1* is mainly expressed at the SCW synthesis period of fiber cells, and GhRLK1 has dual specificity both as a serine/threonine kinase and a tyrosine kinase [79]. All these transcriptome and expression profile implied that RLKs and RLPs might play important roles in fiber development; however, the detailed mechanisms underlying their functions need further study.

Fasciclin-like arabinogalactan proteins (FLAs), a subclass of arabinogalactan proteins, are important for many processes of plant development or adaptation [80]. Huang et al. isolated 19 *GhFLA* cotton genes and showed that *GhFLA1/2/4* were predominantly expressed in 10 DPA fibers, and *GhFLA6/14/15/18* accumulated at relatively high levels [81]. A cotton GPI-anchored lipid transport protein, GhLTPG1, was abundantly expressed in elongating the fibers and the outer integument of the ovules. The knockdown of *GhLTPG1* leads to significantly reduced fiber length, and was due to decreased polar lipid content and repression of fiber elongation-related genes [82]. Phospholipase D (PLD), catalyzes the hydrolysis of phospholipids to produce PA and free polar head groups, and plays diverse roles in plant growth and development [83]. The *GhPLDα1* gene was expressed in various cotton tissues with the highest level in fibers at 20 DPA. The enzyme activity of GhPLDα1 correlated with H_2_O_2_ content and was related to secondary cell wall thickening [84]. The yield and quality of cotton fibers were also significantly affected by reactive oxygen species (ROS) [85], and PM NADPH oxidases (NOXs), also called respiratory burst oxidase homologues (Rbohs), have been shown to be significant sources. There were 13, 13, 26 and 19 Rbohs in *G. arboretum*, *G. raimondii*, *G. hirsutum*, and *G. barbadense*, respectively. Most of these *GhRbohs* were highly expressed in flowers. A few of them were preferentially and specifically expressed during ovule growth and fiber formation, which might be important for fiber development [86]. The *GhCPK1* gene that encodes a PM-localized calcium dependent protein kinase, was primarily expressed in the elongating fiber and might be involved in calcium signaling associated with fiber elongation [87]. Taken together, many of the genes that encodes PM proteins were preferentially expressed in fibers. However, the molecular mechanisms underlying their functions in fiber development need further study.

## 5. Conclusions

Cotton fiber is a highly polarized and elongated single cell, which makes it an ideal model for the study of PM development. Research on cotton fiber development will help us understand the functions of PM in plants. Recently, great progress has been made in cotton fiber development as many important genes have been identified and functionally characterized. The related Genes/Proteins/Reagents and their roles in fiber development are listed in Table 1. However, the regulatory molecular network of fiber cell development is largely unclear. As one of the three major cell systems, the membrane is integral to the regulation of cell growth and development. The major components of the membrane lipid raft (sphingolipids, and sterols), the PM channels, PDs, and PM-resident proteins had all been reported to be involved in cotton fiber elongation or SCW deposition. Further study on the membrane may focus on its role in signal perception and transmission (such as hormone and environmental signaling), protein sorting and transportation, the formation of primary and secondary walls, and cellulose synthesis.

Based on the variety of membrane components, it can be speculated that the function and regulatory mechanism of the membrane system in fiber development are complex. Advances in membrane study could promote research into cotton fiber, and progress in the study of microregions or lipid rafts and advancements in membrane research will shed light on our study. Recently, lipid rafts (lipid microdomains) were considered to be the functional domains of membranes, so lipid raft activity in cotton fiber cells should be given more attention. Additionally, modifying the factors associated with the membrane might disturb the vegetative and reproductive growth of the cotton plant, which is a serious concern because transgenic plants do not produce seed or fiber. Fiber-specific promoters and inducible promoters may reduce the side effects on plant growth, which is conducive to the study of gene function.

## Figures and Tables

**Table 1:** The functions of membrane-related genes in cotton fiber development.

Category	Gene/Protein/Reagent	Expression Profile	Function	References
PM lipids	phosphatidylinositol		promote fiber growth	[23]
	*Δ^15^GhFAD*, *GhPIS*, and *GhPIK*	preferentially in fiber	suppression result in significantly short fiber	[23]
	FB1		severely block fiber elongation	[27]
	VLCFAs		act upstream of ethylene to promote fiber cell elongation	[28]
	*GhDET2*	the initiation stage and rapid elongation stage of fiber	promote fiber number and length	[30]
	*GhSMT2-1*	the rapid elongation stage of fiber	overexpression result in short and thick fiber	[31,33]
PM channels	proton-translocating ATPase, vacuole-ATPase, phosphoenolpyruvate carboxylase, and major intrinsic protein	the period of peak expansion of fiber	involved in turgor regulation	[36]
	PPase	constitutively expressed in fiber		
	*GhPIP1-2* and *GhcTIP1*	predominantly expressed during cotton fiber elongation	supporting the rapid influx of water into vacuoles	[41]
	cotton PIP2 groups		down-regulation lead to markedly hindered fiber elongation	[42]
	*GhWBC1*	highly expressed in developing fiber cells	overexpression result in short siliques in *Arabidopsis*	[44]
	*GhVIN1*	the rapid elongation stage of fiber	promote fiber number and length	[46,47,48]
	sucrose and K+ transporter	consistent with the transient closure of the PDs	related to the osmotic and turgor potentials of fibers	[37]
	*GhGluc1*	high in the short fiber genotypes and weak in the long fiber genotypes	related to PDs closure	[50]
	*Sus*	consistent with the transient closure of the PDs	related to the short fiber cell phenotype of *fls* mutant	[51]
	*GhSCP2D*		reduces plasmodesmal permeability and activates sucrose transporter genes	[52]
PM proteins	*SuSy*	development-dependent expression profiles in cotton fiber	cell wall synthesis	[54,55,56,57,58]
	*GhSusA1*, potato *SuSy*		improve cotton fiber quality	[59,60,61,62]
	*GhCesA4*	SCW synthesis stage	cellulose biosynthesis during cotton fiber development	[66]
	*GhCesA2*			[67]
	*GhCesA1*, *GhCesA2*, *GhCesA7*, and *GhCesA8*		SCW biosynthesis	[68,70]
	*GhCesA3*, *GhCesA5*, *GhCesA6*, *GhCesA9*, and *GhCesA10*		primary cell wall formation	
	*GhCSLD3*		primary cell wall synthesis	[69]
	*AnxGb6*	elongating cotton fibers		[72]
	*AnnGh3*, *AnnGh4*, and *AnnGh5*	preferentially expressed in rapidly elongating fibers		[73]
	*AnxGb5/6*		stabilized scaffold for Actin1 organization	[74]
	*WAK*	highly expressed in cotton fibers and ovules		[78]
	*GhRLK1*	SCW synthesis stage		[79]
	*GhFLA1/2/4*	predominantly expressed in 10 DPA fibers		[81]
	*GhFLA6/14/15/18*	accumulated at relatively high levels in cotton fibers		
	*GhLTPG1*	elongating cotton fibers and outer integument of the ovules	knockdown result in reduction in fiber length	[82]
	*GhPLDα1*	highest level in fibers at 20 DPA	related to secondary cell wall thickening	[84]
	*GhRbohs*	flowers, some expressed in ovules and fibers		[86]
	*GhCPK1*	the elongating fiber	the calcium signaling associated with fiber elongation	[87]

## Data Availability

Not applicable.

## Abbreviations

SCW	secondary cell wall
PM	plasma membrane
PCD	programmed cell death
PDs	Plasmodesmata
CESA	cellulose synthase
PI	phosphatidylinositol
FAD	fatty acid desaturase
PIS	phosphatidylinositol synthase
PIK	phosphatidylinositol kinase
LCB	long chain base
LCFA	long chain fatty acid
FB1	fumonisin B1
Sph	sphinganine
CS	ceramide synthase
DEP	differentially expressed protein
VLCFAs	very-long-chain fatty acids
ACE	2-chloro-N-[ethoxymethyl]N-[2-ethyl-6-methyl-phenyl]-acetamide
ACO	1-aminocyclopropane-1-carboxylic acid oxidase
BR	brassinosteroid
DPA	days past anthesis
AQP	aquaporin
PPase	pyrophosphatase
PIP	plasma membrane intrinsic protein
TIP	tonoplast intrinsic protein
NIP	NOD26-like intrinsic protein
SIP	small basic intrinsic protein
XIP	X intrinsic protein
ABC	ATP-binding cassette
VIN	vacuolar invertase
SuSy	sucrose synthase
GA	gibberellin acid
CSC	cellulose synthase complex
Ces	cellulose synthase
Csl	cellulose synthase-like
NIL	near-isogenic line
TMs	transmembrane helices
GT	glycosyltransferase
RLK	receptor-like kinase
RLP	receptor-like protein
DEG	differentially expressed gene
WAK	wall-associated kinase
FLA	fasciclin-like arabinogalactan protein
GPI	glycosylphosphatidylinositol
PLD	Phospholipase D
ROS	reactive oxygen species
NOXs	NADPH oxidases
Rbohs	respiratory burst oxidase homologues
